# 1292. Activity of Omadacycline and Comparators Against 876 Bacterial Clinical Isolates from Patients with Bone and Joint Infections in the United States and Europe (2015–2022)

**DOI:** 10.1093/ofid/ofad500.1131

**Published:** 2023-11-27

**Authors:** Michael D Huband, Michael A Pfaller, Kelley Fedler, Helio S Sader, Mariana Castanheira

**Affiliations:** JMI Laboratories, North Liberty, Iowa; JMI Laboratories, North Liberty, Iowa; JMI Laboratories, North Liberty, Iowa; JMI Laboratories, North Liberty, Iowa; JMI Laboratories, North Liberty, Iowa

## Abstract

**Background:**

Omadacycline (OMC) is a third-generation tetracycline class (aminomethylcycline) antibacterial with activity against bacterial isolates expressing common tetracycline, penicillin, fluoroquinolone, macrolide, and vancomycin resistance mechanisms. This study determined the *in vitro* activity of OMC and comparators against bacterial clinical isolates collected from patients with bone/joint infections (BJI) from the OMC surveillance program (2015–2022).

**Figure:**
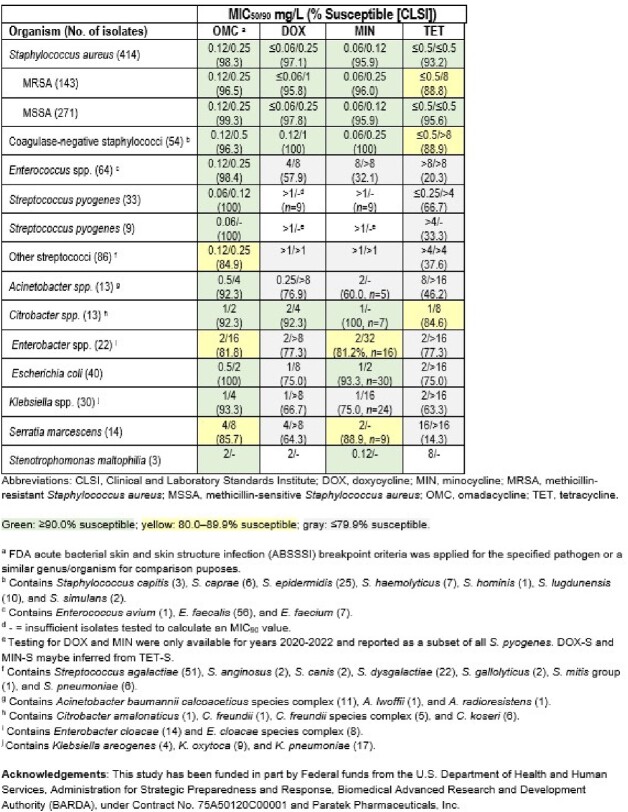

**Methods:**

876 bacterial isolates from bone/joint infections were received from 39 medical centers in the USA and 16 European countries (2015–2022). Identifications were confirmed by MALDI-TOF MS. The top BJI pathogen was staphylococci (53.4% of all isolates), including *S. aureus* (47.3%). Other organism groups included streptococci (13.6%), enterococci (7.5%), and Enterobacterales (18.5%). MIC testing of OMC and comparators was conducted according to CLSI M07 (2018) and M100 (2023) guidelines. MIC results were interpreted using FDA or CLSI breakpoints.

**Results:**

OMC demonstrated potent *in vitro* activity against *S. aureus* (MIC_50/90_, 0.12/0.25 mg/L; 98.3% susceptible [S]) isolates from BJI, including MRSA (96.5%S) and coagulase-negative staphylococci (MIC_50/90_, 0.12/0.5 mg/L; 96.3%S) (Table). All (100%) *S. pyogenes* (MIC_50/90_, 0.06/0.12 mg/L), 98.4% of *Enterococcus* spp. (MIC_50/90_, 0.12/0.25 mg/L), and 84.9% of other streptococci (MIC_50/90_, 0.12/0.25 mg/L) were S to OMC, whereas comparator agent susceptibilities ranged from 20.3% to 57.9%S. Against Gram negatives, ≥90.0% of *Acinetobacter* spp. (MIC_50/90_, 0.5/4 mg/L; 92.3%S), *Citrobacter* spp. (MIC_50/90_, 1/2 mg/L; 92.3%S), *E. coli* (MIC_50/90_, 0.5/2 mg/L; 100%S), *Klebsiella* spp. (MIC_50/90_, 1/4 mg/L; 93.3%S), and *S. maltophilia* (MIC_50_, 2 mg/L; 100%S) isolates were S to OMC (FDA breakpoints applied to similar organism groups for comparison). Comparator agent susceptibilities ranged from 46.2%S to 100%S against these organism groups.

**Conclusion:**

OMC demonstrated potent *in vitro* activity against staphylococci, streptococci, enterococci, and Gram-negative isolates from bone/joint infections, with activity generally more potent than or equal to the other tetracycline class comparator agents.

**Disclosures:**

**Michael D. Huband, BS**, BARDA: This study has been funded in part by BARDA under Contract No. 75A50120C00001.|Entasis: Grant/Research Support|Paratek: Grant/Research Support|Pfizer: Grant/Research Support **Michael A. Pfaller, MD**, Paratek: Grant/Research Support **Kelley Fedler, BS**, Melinta: Grant/Research Support|Paratek: Grant/Research Support **Helio S. Sader, MD, PhD, FIDSA**, AbbVie: Grant/Research Support|Basilea: Grant/Research Support|Cipla: Grant/Research Support|Paratek: Grant/Research Support|Pfizer: Grant/Research Support|Shionogi: Grant/Research Support **Mariana Castanheira, PhD**, AbbVie: Grant/Research Support|Basilea: Grant/Research Support|bioMerieux: Grant/Research Support|Cipla: Grant/Research Support|CorMedix: Grant/Research Support|Entasis: Grant/Research Support|Melinta: Grant/Research Support|Paratek: Grant/Research Support|Pfizer: Grant/Research Support|Shionogi: Grant/Research Support

